# The effects of subjective and objective socioeconomic status on older adults’ mental health in China: a life course-based perspective

**DOI:** 10.3389/fpubh.2026.1789354

**Published:** 2026-04-08

**Authors:** Yu Lin, Bin Cheng, Yun Song, Xuefang Zhuang

**Affiliations:** 1 School of Architecture and Urban Planning, Guangdong University of Technology, Guangzhou, China; 2 School of Architecture and Urban Planning, Chongqing University, Chongqing, China

**Keywords:** older adults, life course, mental health, sex heterogeneity, socioeconomic status

## Abstract

**Introduction:**

China has experienced unprecedented population aging and socioeconomic changes that have raised important questions regarding the determinants of life course strengths and weaknesses in health later in life. Studies have focused on the effect of childhood socioeconomic environments on older adults’ mental health. However, the mechanism of this impact remains unclear, and research on the effects of objective and subjective socioeconomic status on mental health is limited.

**Methods:**

This study used the China Labor-force Dynamic Survey (CLDS) dataset from 2018 and SEM to comprehensively examine the mechanism underlying the effect of socioeconomic status on the mental health of older adults in China from the life-course perspective. This study has systematically compared various dimensions of socioeconomic status, using both objective and subjective measures, and explored gender-based disparities on older adults’ mental health.

**Results:**

The results indicate that childhood socioeconomic status influences mental health through the mediating effect of older adults’ socioeconomic status. A high childhood objective socioeconomic status promotes mental health through the mechanism of older adults’ objective socioeconomic status, supporting the theories of social causation and economic choice. Moreover, childhood subjective socioeconomic status mediates the relationship between childhood objective socioeconomic status and older adults’ socioeconomic status. Furthermore, there is a noticeable difference in the impact of socioeconomic status on mental health between males and females, with the transmission of objective and subjective socioeconomic status being more significant among men and women, respectively.

**Discussion:**

The findings of this study underscore the importance of early interventions targeting socioeconomic status factors across the lifespan to maximize the prevention benefits and improve mental health among older adults in China.

## 1 Introduction

China faces a rapidly aging population, with the proportion of people aged 60 and above projected to increase from 15% in 2015 to 30% by 2050 (1). In this context, the mental health of Chinese older adults poses a serious challenge to the healthcare system and has become a key public health issue (2). Phillips et al. (3) found that the prevalence of mental disorders in the Chinese population was 17.5%, and of these, only 5% had visited a mental health professional. Lei (4) indicated that nearly two-fifths of older adults in China reported sub-clinical levels of depression. Moreover, late-life depression can lead to cognitive impairment, physical decline, and suicide (5). The Healthy China 2030 report notes that society should insist on the comprehensive “full-cycle protection of people’s health” (6), which means that the influencing factors of health in old age should be recognised and researched. It suggests that not only older adults’ current status influences their health, but their early age experiences and behaviors may also have profoundly lasting impacts on their old age health.

Socioeconomic status (SES) has been described as “fundamental to health inequalities,” and these inequalities are not limited by time or space (7). Several studies have demonstrated that SES is important in the persistence of unequal health outcomes (8, 9). For instance, socioeconomically disadvantaged individuals are at greater risk of physical and mental illness in adulthood, leading to health inequities (10). Furthermore, the influences between SES and inequalities are bidirectional. On one hand, the social causation theory suggests that health inequalities arise due to the causal relationship between various health-related factors, such as living and working conditions, access to health services, and social relationships (11, 12). On the other hand, the social selection theory suggests that people with pre-existing conditions drift down the social scale, and those with poor health have a lower SES due to decreased labor force participation or opting out of paid work, which lowers their income and inhibits wealth accumulation (13).

Recently, there is a growing interest in the literature in exploring relationship between childhood SES and health inequalities in adulthood. An extension of the classical status attainment model treats adult health as the presence of cumulative effects from social status (14) and intergenerational transmission (15). Life course approaches are therefore necessary to understand social differences in health, especially on the health relationships between childhood and adult SES (16). Several findings have revealed that childhood SES is an important determinant of adult health, and that people from lower socioeconomic backgrounds are less healthy (17, 18). However, empirical studies on the effect of SES on the mental health of older adults and the transmission effects between socioeconomic groups are scarce, and their findings are mixed. For example, Gilman et al. (19) suggest that childhood SES predicts mental health in adulthood, and social inequalities regarding depression may originate early in life. However, they have also found that childhood SES is unrelated to SES and depression later in life (20). Other studies reported a weak association, largely explained by individuals’ realisation of their childhood SES (21, 22). This suggests that childhood SES determines living and working conditions in adult life and then contributes to social inequalities in mental health. As these studies varied in terms of sample selection and geographic coverage, drawing conclusions about the relationship between childhood and adulthood SES is challenging.

Additionally, in the current literature SES is often measured only through either objective socioeconomic status (OSS) or subjective socioeconomic status (SSS), which may be insufficient for further study base on life course approaches. For older adults, SSS may reflect the cumulative impact of their SES across the lifespan, which is often difficult to measure (23). For instance, educational and occupational statuses may not adequately reflect the social status of older women who have had few educational and occupational opportunities throughout their lives (24). Thus, for older adults’ health study with life course approaches, further utilization of both OSS and SSS is needed. At the same time, previous studies have shown that there are gender differences in socioeconomic status (25). Compared to males, inequality in socioeconomic status is higher and more strongly related to health status among females (26). Exploring gender differences and highlighting specificities or similarities between men and women could help understand mechanisms underlying socioeconomic disparities in mental health, identify effective targets to act on to close the gap between socioeconomic groups and improve the general health of the older adults (27).

Lastly, exploring the relationship between childhood SES and health in later life in China may have extra contribution to this topic due to China’s great social and economic shift in recent decades. Before its “reform and opening-up policy” in 1978, China had been an extremely underdeveloped country with widespread poverty and malnutrition. The market economy reform in 1978 not only improved Chinese people’s general quality of life significantly, but also changed the way of life (28). Individuals born between 1945 and 1978, who were exposed to unfavourable socioeconomic conditions in their early years, are likely to have had a much higher standard of living in late adulthood (29). However, this achievement has not benefited everyone, and concerns regarding the equitable distribution of health benefits from economic progress are growing (30). Meanwhile, China is undergoing a transition from a “rural society” to a “migrant society”, resulting in a substantial number of older adult migrants. According to the China Migrant Population Development Report 2023, the older adult migrant population exceeds 18 million, accounting for 7.2% of the total population in mainland China. Nearly 70 % of these are “older adults drifters” who relocate to support their families or join relatives. Influenced by changes in both physical and social environments, older adult migrants often face numerous challenges in adapting to a new place of residence in later life (31). They commonly experience heightened loneliness, a loss of belonging, and diminished security, which increase their risk of depression (32), particularly for those migrating from rural to urban areas. China, as a comprehensive dual system encompassing economic, political, and social dimensions, exhibits significant disparities in residents’ income levels during its rapid urbanization process. Inequalities in education have widened, reflected in the growing gap in higher education attainment between rural and urban residents (33). Medical service resources, such as hospitals and beds, are unevenly distributed, and health literacy levels are imbalanced between urban and rural areas. Rural residents continue to have lower literacy in health knowledge, behaviors, and skills. The flow of essential resources between urban and rural settings is inefficient, leading to the increasing dominance of cities and a concurrent decline in many rural areas. In addition, against the backdrop of large-scale inter-city migration, persistently low fertility rates, and rising female labor force participation, the geographical distance between older adults who do not migrate for retirement and their adult children is widening, resulting in reduced care and support from their children. These issues inevitably heighten feelings of relative deprivation and abandonment among older adults (34) and socioeconomic status such as income and education has a significant impact on the health of older adults (35). Residents with lower economic status are more susceptible to depressive symptoms (36), contributing to disparities in health status across urban and rural regions.

Thus, despite existing research on the mechanisms of the effect of SES on health, there are still several gaps in the literature that this paper aims to study. First, existing investigations of the effect of SES on older adults’ mental health have paid insufficient attention to the transmission effect among different life stages. Second, most existing studies only measured SES using OSS or SSS, ignoring the interaction effect between OSS and SSS. Third, as sex heterogeneity is observed among the ageing population, estimating sex disparities in the effect of SES on mental health could promote the implementation of “Healthy China 2030” from a Chinese perspective. To fill these gaps, this study examined the factors influencing mental health among older adults in China from a life-course perspective. We posed the following research questions: (1) Does older adults’ SES mediate the effect of childhood SES on the mental health of older adults? (2) What is the relationship between OSS and SSS in the effects of SES on older adults’ mental health? (3) Do the effects of SES on older adults’ mental health differ by gender? The results of this study could shed light on the health risk factors throughout the life course and maximize the benefits of prevention. Furthermore, the results could help understanding population mental health and health inequalities, and provide suggestions regarding health equity policies, and the realization of a healthy China.

## 2 Literature review

### 2.1 Objective socioeconomic status, subjective socioeconomic status, and mental health

**Figure 1:**
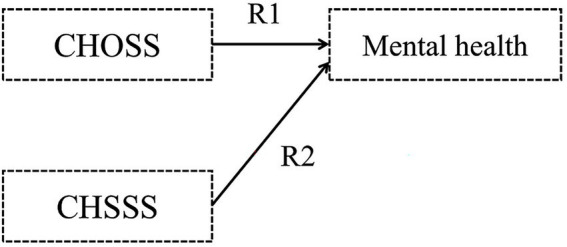
Hypothetical model I.

Objective socioeconomic status (OSS) refers to an individual’s actual relative position in a given social environment and is generally measured using such indicators as educational attainment, employment status, and economic income (37). Research on the relationship between OSS and health dates back to the 1950s. Most early studies focused on the role of structural and material factors, as OSS affects individual health through income level, access to health care, and living environment (38). Recently, the influence of OSS on health has expanded to include lifestyle and psychological factors. For instance, lower OSS affects health primarily through social factors (e.g., relative deprivation of physical and social capital) (39), psychological factors (e.g., lower core self-evaluations with a sense of a future loss of control) (40), and lifestyle (e.g., unhealthy exercise behaviors) (41). This affects levels of mental health. In addition to the traditional measures of social status, researchers have recently focused on subjective socioeconomic status (SSS) as a strong predictor of health (42, 43). SSS is defined as “an individual’s perception of his or her place in the social hierarchy” and considered a new indicator of SES or social class (44), which is commonly calculated by the MacArthur Subjective Social Status Scale (45). Compared to traditional socioeconomic indicators, SSS correlates strongly with health indicators (46), as it is a comprehensive socioeconomic indicator of social status and may capture intangible factors that influence health (47, 48). Based on existing research and the social causation theory, we propose the following hypotheses:

Hypothesis 1: Childhood objective and subjective socioeconomic statuses (CHOSS and CHSSS) impact mental health, respectively (Figure 1).

**Figure 2:**
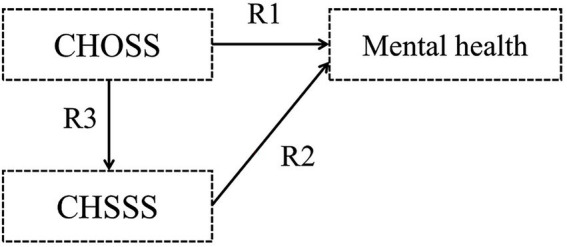
Hypothetical model II.

Hypothesis 2: The CHOSS has an indirect influence on older adults’ mental health, with mediated by the CHSSS (Figure 2).

**Figure 3:**
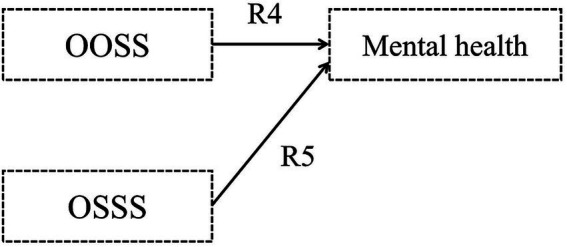
Hypothetical model III.

Hypothesis 3: Older adults’ objective and subjective socioeconomic statuses (OOSS and OSSS) impact mental health, respectively (Figure 3).

**Figure 4:**
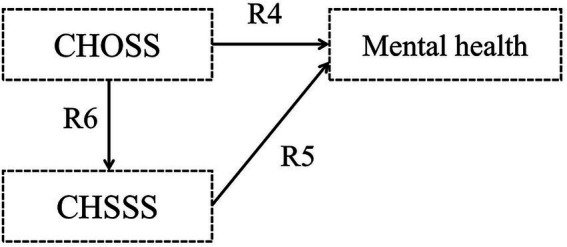
Hypothetical model IV.

Hypothesis 4: The OOSS has an indirect influence on older adults’ mental health, with mediated by the OSSS (Figure 4).

### 2.2 Childhood socioeconomic status and older adults’ mental health

Life course theory suggests that the trajectory of an individual’s life is rooted in the socio-historical period and geographic space in which it is experienced (49). Recently, scholars have focused on the relationship between childhood SES, such as early parental injuries, marital status, and education, and mental health in later life from the life-course perspective to explore health disparities at their source (50–52). The analytical framework includes a critical period, cumulative disadvantage, and pathway models. The critical period model refers to the fact that exposure to risk factors at critical stages of life can sustain long-term effects on the structure and function of organs and systems not altered by later experiences (53). The pathway model suggests that various interrelated exposures influence health later in life, emphasising that risk factors early in life can indirectly influence health in old age through mediating variables. The main pathways are as follows: childhood SES influences health in old age by influencing childhood health (54), childhood SES influences health in old age by influencing OOSS (55), and childhood SES affects childhood health, which affects OOSS, subsequently affecting adulthood (56). Thus, childhood SES affects old-age health both directly and indirectly. However, empirical literature has revealed that the effect of childhood SES on late-adulthood health is mainly indirect. For example, Mckenzie and et al. (55) found that OOSS mediated the effect of childhood SES on mental health in adults.

**Figure 5:**
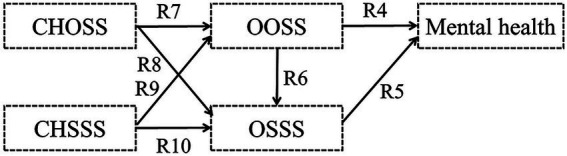
Hypothetical model V.

Economic selection and social causation theories explain these indirect pathways. The economic selection hypothesis emphasises the accumulation of disadvantage and depicts a path dependence process to argue that an initial disadvantage tends to lead to subsequent disadvantages, resulting in a locked-in life trajectory (57). The social causation hypothesis suggests that an individual’s economic status determines their health (57). Poor living conditions and a negative self-consciousness resulting from economic hardship lead to social stressors that continue to undermine mental health (58). Thus, childhood SES may pass through older adults’ SES, affecting mental health based on the economic choice hypothesis and social causation theory. Therefore, we propose the following hypotheses:

Hypothesis 5: Childhood SES, including childhood objective socioeconomic status (CHOSS) and childhood subjective socioeconomic status (CHSSS) indirectly impacts older adults’ mental health through the mediating effect of older adults’ SES, including OOSS and OSSS (Figure 5).

**Figure 6:**
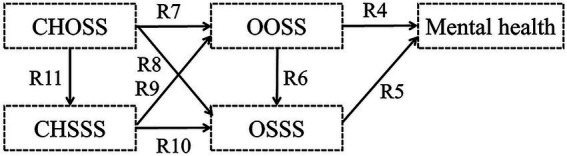
Hypothetical model VI.

Hypothesis 6: A correlation exists between CHOSS and CHSSS, which impacts older adults’ mental health through the mediating effect of older adults’ SES, including OOSS and OSSS (Figure 6).

**Figure 7:**
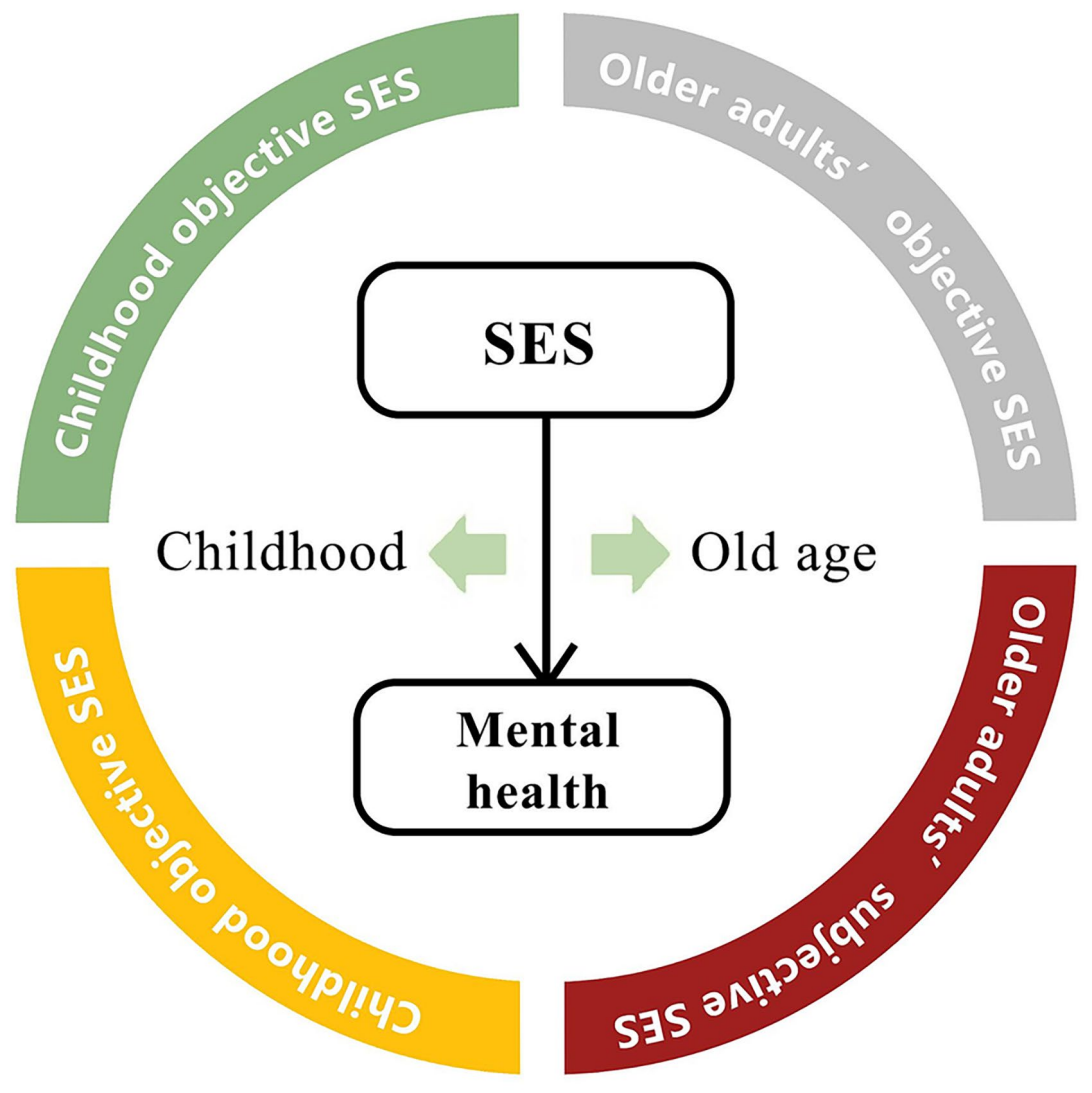
Conceptual framework. SES, socioeconomic status.

Furthermore, this study proposed a conceptual framework of the mechanism underlying the roles of both the objective and subjective socioeconomic status on older adults’ mental health from the life-course perspective, and tries to wake up and confirm it in the following analysis (Figure 7).

## 3 Data and methods

### 3.1 Study area and population

Data are obtained from the 2018 China Labor Dynamics Survey (CLDS), a large-scale, national representative tracking survey of labor force dynamics designed and implemented by the Centre for Social Science Research at Sun Yat-sen University. The 2018 CLDS database consists of data collected from 28 provinces in China, excluding Hong Kong, Macao, Taiwan, Tibet, Hainan, and Xinjiang. The database covers comprehensive data on 368 communities, 13,501 households, and 16,537 individuals in the labor force. In terms of sampling methodology, the 2018 CLDS adopted a multi-stage, multi-level probability sampling method proportional to the size of the labor force, which minimizes sampling errors and ensures the randomness and scientific nature of the sample selection.

This study draws on existing research (59) and defines older adults as individuals aged 60 or older. This study collects 2,932 valid samples from 144 cities in China.

### 3.2 Measurement of variables

#### 3.2.1 Mental health

The CES-D20 Depression Scale is used to measure mental health. The Centre for Epidemiologic Studies Depression Scale contains 20 items for assessing depressive symptoms (60). The scale is scored on a four-point reverse scale (1 = almost always, or 5–7 days per week; 2 = often, or 3–4 days per week; 3 = rarely, or 1–2 days per week; and 4 = almost never, or less than 1 day per week). The total score ranges from 20 to 80, with higher scores indicating an improvement in older adults’ mental health from the previous week. The Cronbach’s *α* for the mental health subscale was 0.950, indicating that this research model passed the reliability test.

#### 3.2.2 Older adults’ objective socioeconomic status and childhood objective socioeconomic status

Based on existing research (61), this study uses older adults’ current educational attainment and annual personal income as measures of the OOSS. CHOSS is generally measured using the SES of parents when the respondents were age 14 (62). We draw on existing research (10, 63, 64) and dichotomized parental education when the respondents were age 14 because the majority of the respondents’ parents and respondents had no schooling at all. Education level is assessed using a single item: “What is your level of education?” The responses were grouped into two categories, “uneducated” or “educated”, and assigning a value of zero or one, respectively. Likewise, according to a large national population-based survey among Danish adults (65), annual income is categorized as “below average” or “equal to or above average” and assigned a value of zero or one, respectively. The average level is the total income of all respondents divided by the total number of respondents.

Furthermore, regarding the measurement of CHOSS (66, 67), participants are asked, “What were your father’s and mother’s educational levels when you were 14 years old”? Responses are grouped into two categories: “uneducated” and “educated” and assigned a value of zero or one, respectively. Previous studies have used parental occupation (55) and place of origin (63) to measure CHOSS. As this study uses the 2018 CLDS database, questions on respondents’ parents’ occupation and place of birth at 14 years of age were not available. Therefore, they were not considered in this study.

#### 3.2.3 Older adults’ subjective socioeconomic status and childhood subjective socioeconomic status

This study uses the MacArthur scale to measure SSS (45). From the previous works on the SSS (68–70), SSS measured by the MacArthur scale has been shown to predict physical health and self-rated health reliably. Furthermore, evidence suggests that single indicator measures may provide more robust and nuanced results than composite measures (71). We also consider that using a single indicator to measure SSS is a limitation of this study. The measurement of a single indicator may not fully reflect the multi-dimensional characteristics of SSS. Participants are asked to indicate their position in a society on a picture of an upright ladder with 10 rungs—with a score of “1” representing the bottom rung and a score of “10” representing the top rung. The OSSS and CHSSS are measured using the questions, “Where do you currently see yourself in the hierarchy?” and “Where did you think your family was in the hierarchy when you were 14 years old?”, respectively. Higher scores indicated higher perceived socioeconomic status.

#### 3.2.4 Covariates

Based on existing research (10, 72), this study adjusts for covariates of sociodemographic and individual health characteristics of older adults’ mental health. For individual-level covariates, this study uses marital status (binary variable: not single vs. single) and hukou (binary variable: local vs. non-local) (i.e., whether the place of domicile was the same as their place of residence) as control variables. For covariates of individual health characteristics, this study includes illness and injury status (binary variable: no sickness or injury within the last 2 weeks vs. sickness and injury within the last 2 weeks), history of alcohol consumption (binary variable: no history of alcohol consumption vs. history of alcohol consumption), and history of smoking consumption (binary variable: no history of smoking consumption vs. history of smoking consumption). All the above variables are presented in Table 1.

**Table 1:** Statistics of variables.

Variables	Assignments	Total	Men	Women
Number		2,932	1,563 (53.30%)	1,369 (46.70%)
Mental Health [mean (SD)]	Continuous variables (20–80)	72.03 (9.77)	73.05 (8.82)	70.87 (10.64)
CHOSS				
Father’s education [*N* (%)]	1 = Educated	996 (34%)	558 (35.7%)	438 (32%)
	0 = Uneducated	1936 (66%)	1,005 (64.3%)	931 (68%)
Mother’s education [*N* (%)]	1 = Educated	419 (14.3%)	222 (14.2%)	197 (14.4%)
	0 = Uneducated	2,513 (85.7%)	1,341 (85.8%)	1,172 (85.6%)
CHSSS [mean (SD)]	Continuous variables (1–10)	3.14 (1.72)	3.09 (1.71)	3.21 (1.74)
OOSS				
Income level [*N* (%)]	1 = equal to or above average	1,616 (55.1%)	810 (51.8%)	806 (58.9%)
	0 = below average	1,316 (44.9%)	753 (48.2%)	563 (41.1%)
Educational attainment [*N* (%)]	1 = Educated	2,093 (71.4%)	1,329 (85%)	604 (44.2%)
	0 = Uneducated	838 (28.6%)	234 (15%)	764 (55.8%)
OSSS	Continuous variables (1–10)	4.46 (1.78)	4.44 (1.81)	4.47 (1.76)
Covariates				
Marital status [*N* (%)]	1 = Non-single	2,647 (90.3%)	1,424 (91.1%)	1,223 (89.3%)
	0 = Single	285 (9.7%)	139 (8.9%)	146 (10.7%)
Hukou [*N* (%)]	1 = Local	2,800 (95.5%)	1,494 (95.6%)	1,306 (95.4%)
	0 = Non-local	132 (4.5%)	69 (4.4%)	63 (4.6%)
Sickness and injury status [*N* (%)]	1 = No sickness or injury within the last 2 weeks	2,509 (85.6%)	1,373 (87.8%)	1,136 (83.0%)
	0 = Sickness and injury within the last 2 weeks	423 (14.4%)	190 (12.2%)	233 (17.0%)
History of alcohol consumption [*N* (%)]	1 = No history of alcohol consumption	2,260 (77.1%)	955 (61.1%)	1,305 (95.3%)
	0 = History of alcohol consumption	672 (22.9%)	608 (38.9%)	64 (95.3%)
History of smoking consumption [*N* (%)]	1 = No history of smoking consumption	1955 (66.7%)	655 (41.9%)	1,300 (95.0%)
	0 = History of smoking consumption	977 (33.3%)	908 (58.1%)	69 (5.0%)

SD, standard deviation; CHOSS, childhood objective socioeconomic status; CHSSS, childhood subjective socioeconomic status; OOSS, older adults’ objective socioeconomic status; OSSS, older adults’ subjective socioeconomic status.

### 3.3 Analyses

#### 3.3.1 Structural equation modelling

This study assesses older adults’ mental health through their OOSS, OSSS, CHOSS, CHSSS, individual health status, and sociodemographic characteristics. Several variables affect older adults’ mental health while also influencing each other. For instance, OOSS mediates the relationship between CHOSS and mental health. Therefore, this study used structural equation modelling (SEM) due to its flexibility in examining the relationships between multiple causes and effects. Furthermore, SEM is a powerful multivariate statistical method to explore and test the causal relationships between observed and latent variables (73).


Y=Λyη+ε (1)

X=Λxξ+o (2)

ŋ=Bη+Γξ+ς (3)

Structural models (1) and (2) relate to the indicators and latent variables, whereas structural model (3) relates to the relationship between the latent variables. In these models, ξ is an exogenous latent variable unaffected by any other variable in the model. Typically, an exogenous latent variable affects one or more of a model’s variables. Moreover, η is an endogenous latent variable, which is affected by one or more other variables in the model. An endogenous latent variable may affect another endogenous latent variable in the model. In these equations, Λy and Λx are the factor loadings’ coefficients; B and Г are the path coefficients; and o and *ε* are the measurement errors of the observed variables *x* and *y*, respectively. This study uses CHOSS and OOSS as latent variables.

#### 3.3.2 Conceptual model

Before analysing the SEM results, we have conducted an exploratory factor analysis and selected variables based on *t*-values and factor loadings greater than 0.4. After screening, the latent variable CHOSS consists of two measured variables: the father’s and mother’s education during childhood, the latent variables for OOSS were income level and education. Each measure’s factor loadings are a variable fit, and the adjusted Kaiser-Meyer-Olkin tests are all greater than 0.6, indicating the model’s validity. Most existing studies use three or more observed variables to measure the latent variables. Moreover, when the sample size is greater than 400, the CFA model allows only two indicators for each latent variable (57, 58). Therefore, having two observed indicators to explain the latent variables is acceptable.

## 4 Results

### 4.1 Descriptive statistics

Table 1 presents summary statistics of the variables used in the model. Specifically, men had higher mean mental health scores than women (73.05 vs. 70.87). In terms of childhood SES, the CHSSS scores of men were lower than those of women (3.09 vs. 3.21), and in terms of older adults’ SES, the men’s OSSS scores are lower than those of women (4.44 vs. 4.47). A higher proportion of the men cohort is educated than the women cohort (85.0% vs. 44.2%). In terms of the control variables, a larger proportion of the men have non-single than the women (91.1% vs. 89.3%), and a higher proportion of the men have a local hukou than the women (95.6% vs. 95.4%). In addition, a higher proportion of men than women have no injuries or illnesses (87.8% vs. 83.0%). Smoking and drinking are more prevalent among males than females.

### 4.2 Statistical analysis and model testing

This study uses SEM to create and test six hypothetical models, which are statistically analyzed in Stata 13 software using the maximum likelihood method. Table 2 lists the fit parameters for each of the six models. We have modelled and tested the six hypotheses. Finally, this study considers existing research in measuring the fit parameters for SEM (74) and selecting a path model with relatively well-fitting parameters to explore the heterogeneity between men and women. The following model-fit parameter criteria are used: λ2 test of goodness of fit (λ2/df); root mean square error of approximation (RMSEA) ≤ 0.08; goodness-of-fit index (GFI) ≥ 0.90; normed fit index (NFI) ≥ 0.90; incremental fit index (IFI) ≥ 0.90; and comparative fit index (CFI) ≥ 0.90. These manipulations are performed using SPSS Amos 26.

**Table 2:** Model fit parameters.

Fitted parameter	Model I	Model II	Model III	Model IV	Model V	Model VI
λ^2^/df	5.237	3.928	12.251	5.452	2.709	2.231
RMSEA	0.038	0.032	0.062	0.039	0.035	0.034
GFI	0.990	0.993	0.976	0.991	0.984	0.988
NFI	0.905	0.932	0.724	0.891	0.891	0.926
IFI	0.922	0.948	0.741	0.910	0.929	0.942
CFI	0.921	0.948	0.739	0.909	0.927	0.941

λ^2^/df, λ^2^ test of goodness of fit; RMSEA, root mean square error of approximation; GFI, goodness-of-fit index; NFI, normed fit index; IFI, incremental fit index; CFI, comparative fit index.

**Figure 8:**
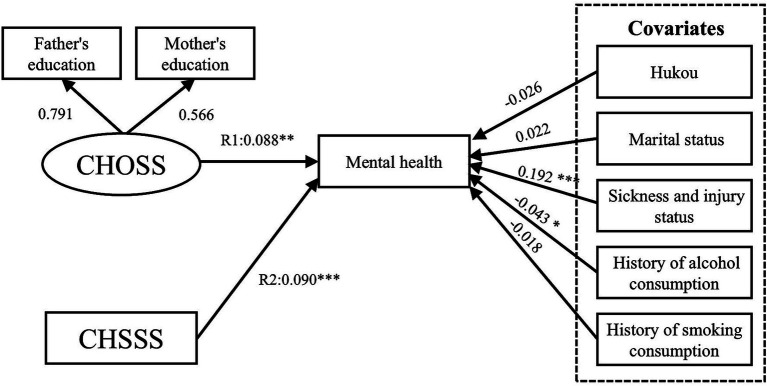
Model I SEM results with standardised coefficients. CHOSS, childhood objective socioeconomic status; CHSSS, childhood subjective socioeconomic status. *p ≤ 0.05, **p < 0.01, ***p < 0.001.

**Figure 9:**
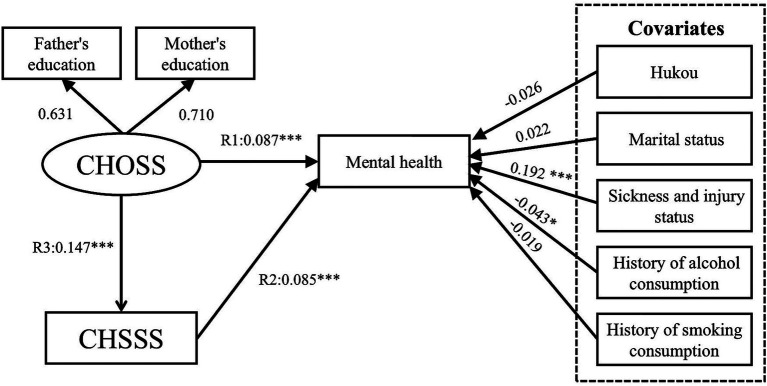
Model II SEM results with standardised coefficients. CHOSS, childhood objective socioeconomic status; CHSSS, childhood subjective socioeconomic status. *p ≤ 0.05, **p < 0.01, ***p < 0.001.

**Figure 10:**
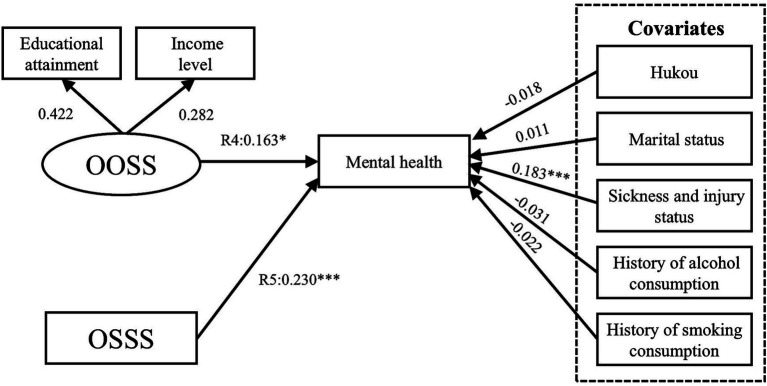
Model III SEM results with standardised coefficients. OOSS, older adults’ objective socioeconomic status; OSSS, older adults’ subjective socioeconomic status. *p ≤ 0.05, **p < 0.01, ***p < 0.001.

**Figure 11:**
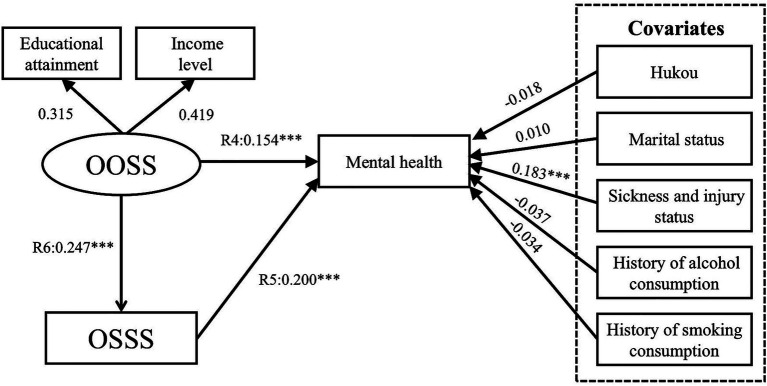
Model IV SEM results with standardised coefficients. OOSS, older adults’ objective socioeconomic status; OSSS, older adults’ subjective socioeconomic status. ^*^p ≤ 0.05, ^**^p < 0.01, ***p < 0.001.

Table 3 shows the direct effects between childhood SES, older adults’ SES and mental health. The results of Model I confirm Hypothesis 1 (Figure 8), indicating that the path of the CHOSS and CHSSS’ effect on mental health was significant, with R1 and R2 values of 0.088 (*p* < 0.01) and 0.090 (*p* < 0.001), respectively. The results of Model II confirm Hypothesis 2 (Figure 9), with path coefficient for R3 of 0.147 (*p* < 0.001) for the CHOSS on the CHSSS. The results of Model III confirm Hypothesis 3 (Figure 10), indicating that the path of the OOSS and OSSS’ effect on mental health was significant, with R4 and R5 values of 0.163 (*p* < 0.05) and 0.230 (*p* < 0.001), respectively. The results of Model IV confirm Hypothesis 4 (Figure 11), with path coefficient for R6 of 0.247 (*p* < 0.001) for the OOSS on the OSSS. The comparison of Models III and IV reveals that the latter has a better goodness of fit. Therefore, Hypotheses 3 and 4 are accepted.

**Table 3:** Standard parameters for direct effects in SEM.

Trails	Total		Men		Women	
	Estimate	Standard error	Estimate	Standard error	Estimate	Standard error
R4: OOSS → Mental health	0.137***	0.027	0.138***	0.04	0.103**	0.038
R5: OSSS → Mental health	0.211***	0.021	0.213***	0.026	0.225***	0.033
R6: OOSS → OSSS	0.164**	0.065	0.153	0.181	0.111	0.068
R7: CHOSS → OOSS	0.635***	0.047	0.657***	0.084	0.611***	0.06
R8: CHOSS → OSSS	−0.071	0.053	−0.108	0.156	0.023	0.06
R9: CHSSS → OOSS	0.114**	0.039	0.137*	0.062	0.084	0.047
R10: CHSSS → OSSS	0.398***	0.023	0.394**	0.05	0.407***	0.032
R11: CHOSS → CHSSS	0.145***	0.025	0.128***	0.035	0.165***	0.034
Covariates						
Hukou → Mental health	−0.016	0.015	−0.039	0.019	0.005	0.022
Marital status → Mental health	0.011	0.019	0.003	0.023	0.012	0.028
Sickness and injury status → Mental health	0.183***	0.021	0.175***	0.029	0.188***	0.029
History of alcohol consumption → Mental health	−0.035*	0.018	−0.025	0.024	0.018	0.028
History of smoking consumption → Mental health	−0.030	0.019	0.011	0.025	0.048	0.031

CHOSS, childhood objective socioeconomic status; CHSSS, childhood subjective socioeconomic status; OOSS, older adults’ objective socioeconomic status; OSSS, older adults’ subjective socioeconomic status.

*p ≤ 0.05, **p < 0.01, ***p < 0.001.

**Figure 12:**
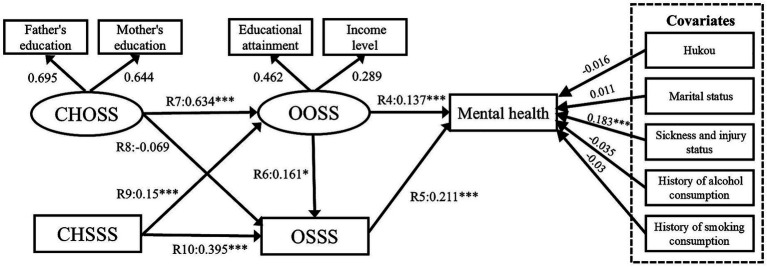
Model V SEM results with standardized coefficients. CHOSS, childhood objective socioeconomic status; CHSSS, childhood subjective socioeconomic status; OOSS, older adults’ objective socioeconomic status; OSSS, older adults’ subjective socioeconomic status. *p ≤ 0.05, **p < 0.01, ***p < 0.001.

**Figure 13:**
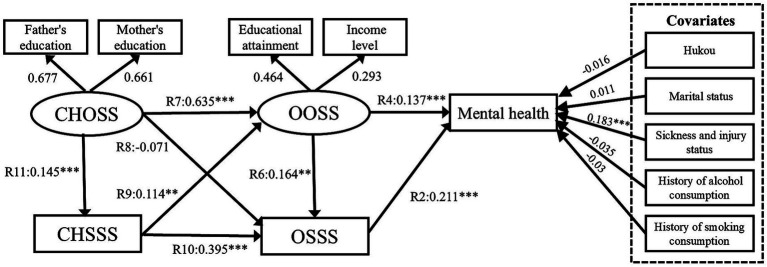
Model VI SEM results with standardized coefficients. CHOSS, childhood objective socioeconomic status; CHSSS, childhood subjective socioeconomic status; OOSS, older adults’ objective socioeconomic status; OSSS, older adults’ subjective socioeconomic status. *p ≤ 0.05, **p < 0.01, ***p < 0.001.

Model V results confirmed Hypothesis 5 (Figure 12). The path coefficients R4, R5, and R6 are 0.137 (*p* < 0.001), 0.211 (*p* < 0.001), and 0.161 (*p* < 0.05), respectively. The path coefficients R7, R9 and R10 are 0.634 (*p* < 0.001), 0.15 (*p* < 0.001), and 0.395 (*p* < 0.001), respectively. The R8 path is not significant (*p* > 0.05). The Model VI results confirmed Hypothesis 6 (Figure 13). The path coefficient R11 is 0.145 (*p* < 0.001). The comparison of Models V and VI indicates that the model fitness values of Model VI are satisfactory and better than those of Model V (75). Moreover, Model VI better explains the relationship between the five factors (CHOSS, CHSSS, OOSS, OSSS, and mental health). Thus, this study accepts Model VI and confirms our conceptual framework (Figure 7).

### 4.3 Potential relationships between childhood socioeconomic status and mental health

Table 4 presents CHOSS’ indirect effects on older adults’ SES and mental health. To examine the mediation effects, we investigated significant interrelationships using maximum likelihood with 2,000 bootstrap samples at 95% bias-corrected confidence intervals (76). We examined the significance of the indirect effects by calculated confidence intervals (CIs) for both lower and upper bounds (77) in Table 5. The relationships between CHOSS and OOSS are both fully mediated effects of “CHOSS - OOSS – OSSS”, “CHOSS - CHSSS – OSSS”, and “CHOSS - CHSSS - OOSS – OSSS”. Additionally, the CHOSS’ effect on older adults’ mental health could be realised through three mediating variables: CHSSS, OOSS and OSSS. This result suggests that CHOSS largely determined OOSS, which significantly influenced older adults’ mental health. Furthermore, the impact pathway “CHOSS - CHSSS - OSSS - mental health” was established.

**Table 4:** Standard parameters for indirect effects in SEM.

Trails	Total		Men		Women	
	Estimate	Standard error	Estimate	Standard error	Estimate	Standard error
T1: CHOSS → OOSS → OSSS	0.104**	0.047	0.101	0.151	0.068	0.047
T2: CHOSS → OOSS → Mental health	0.087***	0.017	0.091***	0.027	0.063**	0.024
T3: CHOSS → OOSS → OSSS → Mental health	0.022**	0.010	0.021	0.032	0.015*	0.011
T4: CHOSS → OSSS → Mental health	−0.015	0.011	−0.023	0.033	0.005	0.014
T5: CHOSS → CHSSS → OOSS	0.016**	0.006	0.018*	0.01	0.014*	0.008
T6: CHOSS → CHSSS → OOSS → Mental health	0.002**	0.001	0.002*	0.001	0.001*	0.001
T7: CHOSS → CHSSS → OOSS → OSSS → Mental health	0.001**	0.001	0.001	0.001	0.001	0.001
T8: CHOSS → CHSSS → OSSS	0.058***	0.010	0.050**	0.015	0.067***	0.015
T9: CHOSS → CHSSS → OSSS → Mental health	0.012***	0.002	0.011**	0.003	0.015***	0.004
T10: CHOSS → CHSSS → OOSS → OSSS	0.003**	0.002	0.003	0.005	0.002	0.002

CHOSS, childhood objective socioeconomic status; CHSSS, childhood subjective socioeconomic status; OOSS, older adults’ objective socioeconomic status; OSSS, older adults’ subjective socioeconomic status.

*p ≤ 0.05, **p < 0.01, ***p < 0.001.

**Table 5:** Bias-corrected 95% CI for indirect effects in SEM.

Trails	Total		Men		Women	
	Bias-corrected 95% CI		Bias-corrected 95% CI		Bias-corrected 95% CI	
T2: CHOSS → OOSS → Mental health	0.759	7.302	−6.140	6.680	−0.527	6.591
T3: CHOSS → OOSS → OSSS → Mental health	0.230	1.443	−0.278	4.949	−0.003	1.517
T4: CHOSS → OSSS → Mental health	−1.276	0.111	−4.678	0.419	−0.927	1.059
T6: CHOSS → CHSSS → OOSS → Mental health	0.020	0.219	−0.111	0.237	−0.005	0.266
T7: CHOSS → CHSSS → OOSS → OSSS → Mental health	0.004	0.053	−0.004	0.299	0.000	0.064
T9: CHOSS → CHSSS → OSSS → Mental health	0.224	0.550	0.110	0.525	0.293	0.923

CHOSS, childhood objective socioeconomic status; CHSSS, childhood subjective socioeconomic status; OOSS, older adults’ objective socioeconomic status; OSSS, older adults’ subjective socioeconomic status.

### 4.4 Analysis of sex heterogeneity

**Figure 14:**
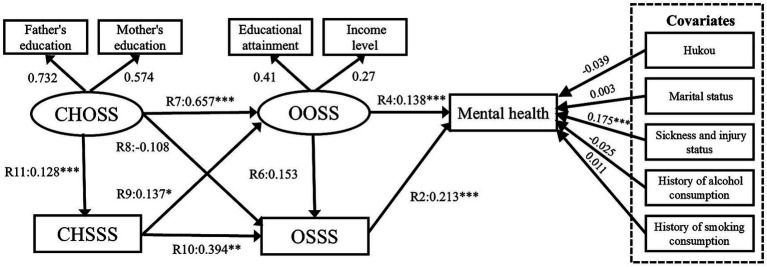
Model VII SEM results with standardized coefficients. CHOSS, childhood objective socioeconomic status; CHSSS, childhood subjective socioeconomic status; OOSS, older adults’ objective socioeconomic status; OSSS, older adults’ subjective socioeconomic status. *p ≤ 0.05, **p < 0.01, ***p < 0.001.

**Figure 15:**
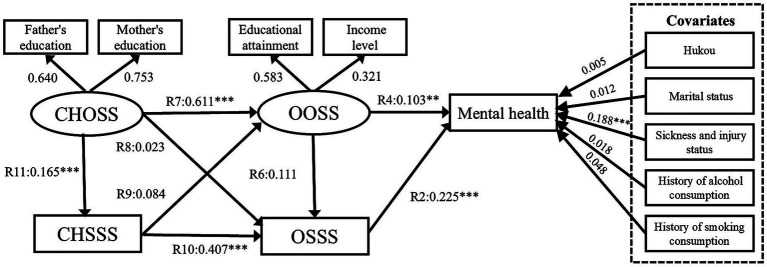
Model VIII SEM results with standardised coefficients. CHOSS, childhood objective socioeconomic status; CHSSS, childhood subjective socioeconomic status; OOSS, older adults’ objective socioeconomic status; OSSS, older adults’ subjective socioeconomic status. *p ≤ 0.05, **p < 0.01, ***p < 0.001.

This study uses pathway Model VI (see Subsection 4.2) to further explore the presence of sex heterogeneity among the factors of childhood SES, older adults’ SES, and mental health. In previous studies (26), we learned about the significance of examining the socioeconomic status of older adults of different genders in relation to their health. Meanwhile, we conducted sub-sample analyses based on urban–rural areas, income, and other factors, and we found differences in gender heterogeneity, so we conducted further research on older adults of different genders. Model VII presents the results for the men sub-sample (Figure 14; λ2/df = 3.493, RMSEA = 0.04, GFI = 0.983, NFI = 0.861, IFI = 0.902, and CFI = 0.904). Model VIII presents the results for the women sub-sample (Figure 15; λ2/df = 2.231, RMSEA = 0.03, GFI = 0.987, NFI = 0.912, IFI = 0.950, and CFI = 0.949).

First, the OOSS’ effect on older adults’ mental health is significant among men sub-sample (R4 = 0.138, *p* < 0.001). In the woman subsample, OOSS’ effect on older adults is significant associated with mental health (R4 = 0.103, *p* < 0.01). This suggests that men and women generally value OOSS, and acquiring OOSS significantly contributes to their mental health. It is worth noting that the CHSSS of males has a significant effect on OOSS (R6 = 0.137, *p* < 0.05), however, there is no evidence that the CHSSS of females has a significant effect on OOSS. In addition, Table 5 presents the “CHOSS – CHSSS – OSSS – mental health” pathways was significant among the male samples. CHOSS can positively affect mental health through CHSSS and OSSS. In the women sample, two mediating pathways are significant, namely “CHOSS – CHSSS – OSSS – mental health” and “CHOSS – CHSSS – OOSS – OSSS – mental health.”

## 5 Discussion

This study adopts a life-course perspective and uses statistics from the national representative 2018 CLDS database. This study uses SEM to construct a path hypothesis model of the impacts of objective and subjective socioeconomic statuses in childhood and old age on the mental health of older adults. Subsequently, this study analyzed the heterogeneity of the mechanism by sex, which could facilitate early interventions for health risk factors throughout the life course. Thus, the results maximize the benefits of health risk prevention and provide lessons to improve older adults’ health and develop health equity policies.

### 5.1 Older adults’ socioeconomic status and mental health

This study confirms that CHOSS and OOSS affect mental health directly and also indirectly through CHSSS and OSSS, which is consistent with a number of existing studies (78, 79). Furthermore, this study demonstrates that geriatric SSS has a great effect on older adults’ mental health. This is because SSS is a comprehensive indicator of a person’s assessment of their relative position in contemporary society, capturing OSS intangibles (48) and psychological processes, such as interpersonal relative deprivation (80). Unlike younger adults, older adults reach the final stage of their lives when they are aware of their social status. Perceived social status reflects OSS’ cumulative impact over their lifetime and their lifelong accomplishments (4, 79). Therefore, perceived social status may influence older adults’ lives and prospects.

### 5.2 Potential pathways of childhood socioeconomic status to older adults’ mental health

The path analysis results for childhood and older adults’ SES reveals that the standardized path coefficient of CHOSS on OOSS have a significant effect. This suggests that SES is characterized by intergenerational transfers, supporting the status acquisition model. Some studies report that individuals who have a stable high SES across the life course typically have better health, while people with a stable low SES typically report the worst health (81, 82) and people living in areas with higher intergenerational mobility often have higher levels of happiness (83). Parents’ education level during childhood affects children’s later education level by influencing their willingness to invest in their children’s education and perceptions. This ultimately realizes the intergenerational transfer of education (84). Furthermore, this study finds that CHOSS does not directly influence OSSS but rather mediates the relationship with OSSS through both CHSSS and OOSS. However, one study has found that CHOSS remained associated with OSSS, even after adjusting for OOSS (85). Older adults are at a late stage in their life course, with the influence of and dependence on the family of origin and current family reduced. Thus, although CHOSS is not strongly associated with OSSS, CHOSS can influenced OOSS (e.g., pensions and social security), subsequently affecting OSSS.

Furthermore, the findings support the “pathways” argument from a life-course perspective, in which social and economic factors early in life (e.g., childhood family income and relative position in society) influence later life experiences, opportunities, and health primarily through indirect socioeconomic pathways, including education (86). Specifically, higher CHOSS is associated with better mental health in old age, with the former influencing the latter through multiple pathways, including the CHSSS, OOSS and OSSS. Consistent with existing research (54, 55, 87), the pathway “CHOSS - OOSS - mental health” suggests that CHOSS has long-term effects on mental health later in life, and these effects persist even if their OOSS improves (88). Notably, education may be an important marker in the transition from the SES acquired from parents to SES realized in adulthood. Education shapes health through its impact on the socioeconomic environment of adulthood, as higher levels of education often bring better jobs, incomes, housing, neighbourhoods, and working conditions (55). Although investing in education can improve an individual’s chances of attaining higher social status in the future (89). In the current society where economic growth is taking place while the income gap is widening even more. Family education investment is negatively affected by income inequality (90). The provision of good opportunities in poor families is reduced, thereby exacerbating overall inequality (91, 92). This indicates that family support usually exerts an influence on social and economic status through the role of education (93). Moreover, CHSSS primarily affects older adults’ mental health by influencing OSSS, which in turn affects older adults’ mental health. This illustrates the relative sense of social deprivation felt in childhood, such as a lack of resources, opportunities, or attention, which may psychologically create a sense of insecurity and lack of self-worth. These feelings may bring a psychological shadow as individuals age. From the perspective of urban and rural areas, studies have shown that residents living in cities have a higher social status. Among them, education and income are the key driving factors (94). Urban children tend to have better socioeconomic status, which may encourage them to achieve higher educational attainment and career success in the future (95).

### 5.3 Sex heterogeneity

This study identifies sex heterogeneity in the childhood SES pathway to mental health. The association between SES and depression is strong among women (96, 97). Although existing literature has identified sex differences in the association between SES and depression, in-depth analyses of differences in the pathways of the association are lacking. This study finds that among men, CHOSS largely influences mental health through influencing OOSS while for women, CHOSS largely influences mental health by influencing CHSSS and OSSS. Women focused on SSS effects on mental health. Women are more dependent on emotional support and relationships in which emotional closeness, trust, and solidarity are exchanged (98). Simultaneously, women take on a “duty of care”, implying that they are exposed to and sensitive to social networking events, and more susceptible to feelings of relative deprivation. This leads to increased levels of depression when experiencing interpersonal stress or the loss of relationships. Conversely, men are prone to depression from stressful work-related events (99). Additionally, financial difficulties in childhood appear to be more significant and have a stronger impact on depression throughout the life course among men than women (100, 101). Men appear to be more likely to experience depression in an absolute sense. Thus, this study shows that policies to address mental health outcomes in adulthood must address childhood SES while considering the varied vulnerabilities of men and women (102).

### 5.4 Subjective variable explaining subjective variable

The findings of our study have suggested significant correlations among childhood and older adults’ subjective social-economic status (CHSSS, OSSS) and their mental health in older age, however as CHSSS, OSSS and mental health are all subjective variables, such explanation may lead to potential problems of confounding bias. In other words, the correlations among these three variables may be driven by underlying psychological traits that jointly shape respondents’ perceptions and self-reports. Thus, such explanations need extra justification through either theoretical reasoning, variable measurement, or data analysis (103).

Here, we believe theoretical reasoning is suitable to justify our findings. Indeed, CHSSS, OSSS, and mental health may be influenced by underlying psychological traits, as those with poor mental health are likely to underestimate their CHSSS and OSSS. However, CHSSS, OSSS and mental health are shaped through different psychological mechanisms. Firstly, mental health status is shaped by one’s physical, psychological and social status, of which its psychological condition is heavily influenced by one’s emotional experiences. Emotional experiences can be dramatically changed in rather short period of time due to sudden specific incidents. For instance, when drastic life changes (e.g., divorce, loss of a child) occurred, older adults may experience great emotional impact such as extreme sadness and mental instability, which can significantly affect their mental health. Thus, the psychological mechanism that shapes one’s mental health is mainly through emotional experiences.

On the other hand, subjective social-economic status measures people’s self-evaluation regarding their own position within the social hierarchy, namely social perception with objective foundation. Both CHSSS and OSSS are subjective assessments grounded in objective facts. Which are relatively stable and do not easily influenced by sudden life incidents and emotional experiences. For instance, one who grew up in a well-off family is not likely to underestimate their objectively privileged childhood just because the current emotional feeling. Similarly, those in a good mood is unlikely to overestimate their social status if they do not have objective bases. Therefore, CHSSS and OSSS may be somewhat influenced by psychological status to some extent, but the impact is limited, and they have rather different psychological mechanisms comparing to mental health. Therefore, it is justified to explain one’s mental health through CHSSS and OSSS measurements.

### 5.5 Limitations

This study had several limitations. First, this study uses retrospective self-reporting, which may be subject to recall bias. Second, this study focuses on the transmission effects of SES on mental health over time reflecting social causation and economic choice theory, however, this study does not discuss the role of social choice theory (104) in health. We have tried to use theoretical reasoning to handle potential problems of confounding bias in the above sections: poor mental health may bias individuals’ perceptions, causing them to underestimate their childhood SSS and OSSS. However, this strategy also has limitations. It does not directly test the data and cannot completely eliminate the confounding effects of potential psychological traits. Thus, future research should employ longitudinal data and utilize the instrumental variable method to mitigate the impact of endogeneity issues.

## 6 Conclusion

This study demonstrates that the influence of CHOSS on older adults’ mental health is mainly realised through the mediating variables of CHSSS, OOSS and OSSS. Furthermore, the mediating variables intertwined and influenced each other. Second, CHOSS determines OOSS, which is the main influencing factor of mental health in old age. The results of this study support the social causation and economic choice theories. It suggests that one’s childhood social economic status does has an impact on his/her mental health in old age, especially objective social economic status can be inherited from their parents and influence people for a lifetime, even to their mental health in old age.

Moreover, sex heterogeneity is observed in the influence of SES on older adults’ mental health, with women exhibiting a transmission effect between CHSSS and OSSS and men between the CHOSS and OOSS. The mental health of men is affected by objective socioeconomic status likely because men still have dominated wealth and physical recources both in family and society in the Chinese context, while the typical role for women is still about family and relationship therefore their mental health in old age is linked closer with subjective factors. This study has policy implications for building a healthy ageing society. The government should provide “precise assistance” to children of low socioeconomic status from the early stages of life, and promote the accumulation of human capital in the older adults population by vigorously developing education and vocational training. In addition, the government should build an institutional mechanism to promote upward social mobility, eliminate the sense of deprivation of social classes, break down institutional barriers, strengthen psychological identity, and enhance the subjective social identity of the older adult population. Physical and mental health are prerequisites for older adults’ participation in society and form the foundation for ensuring their quality of life in later years. Given the accelerating trend of population aging and the reality of China’s urban–rural dual structure, health inequalities among the older adults warrant urgent attention. Strengthening societal care and support for older adults can help prevent suicide and other complications, holding practical reference value for achieving the goals of Healthy China and advancing the implementation of the healthy aging strategy.

## Data Availability

Publicly available datasets were analyzed in this study. This data can be found at: http://css.sysu.edu.cn; cssdata@mail.sysu.edu.cn.
